# A Biometric Comparison Between Myopic and Non-myopic Eyes Treated for Retinopathy of Prematurity

**DOI:** 10.18502/jovr.v20.14953

**Published:** 2025-05-05

**Authors:** Kaveh Abri Aghdam, Samira Chaibakhsh, Nazanin Hasani, Vahid Zare Hosseinabadi, Ali Aghajani

**Affiliations:** ^1^Eye Research Center, The Five Senses Health Institute, Department of Ophthalmology, School of Medicine, Iran University of Medical Sciences, Tehran, Iran; ^2^Rajaie Cardiovascular Medical and Research Institute, Iran University of Medical Sciences, Tehran, Iran; ^3^Isfahan Eye Research Center, Department of Ophthalmology, Isfahan University of Medical Sciences, Isfahan, Iran

**Keywords:** Myopia, Ocular Biometry, Refraction, Retinopathy of Prematurity

## Abstract

**Purpose:**

This study aims to assess the biometric alterations contributing to myopia in children who have undergone treatment for retinopathy of prematurity (ROP) and compare these changes with those observed in full-term myopic children.

**Methods:**

Children who had undergone ROP treatment were recruited and classified according to their treatment methods. An age-matched group of myopic patients with no history of ROP treatment was also included. Complete perinatal history was collected, and a comprehensive ophthalmic examination, including cycloplegic refraction, was conducted. The biometric data of children in each study group were gathered using the IOL Master and Pentacam.

**Results:**

The study recruited 14 patients in the intravitreal bevacizumab (IVB) group, 17 patients in the laser-treated group, and 13 individuals in the control group. There was no significant difference between the two patient groups regarding gestational age, birth weight, and age. In the IVB group, 50% of patients were myopic, compared to 52.9% in the laser-treated group. The incidence of high myopia was significantly higher in the laser-treated group (*P* < 0.001). In the non-myopic group, changes in refractive error were solely related to changes in axial length (*P *= 0.003). However, in the myopic group, changes in refractive error were significantly associated with changes in anterior chamber depth (*P* < 0.001), lens thickness (*P* < 0.001), and axial length (*P *= 0.018). Furthermore, myopic children in the ROP group had significantly shorter axial lengths, shallower anterior chambers, thicker lenses, and steeper corneas compared to the control group (all *P* < 0.001).

**Conclusion:**

Eyes with a history of ROP treatment, whether myopic or non-myopic, should be considered distinct entities. In patients who have undergone ROP treatment and have not developed myopia, changes in refractive error are primarily influenced by alterations in axial length, rather than changes in the anterior segment. Furthermore, children with myopia and a history of treatment for ROP (either IVB or laser) exhibit different biometric changes compared to myopic children without a history of ROP treatment, further underscoring their unique characteristics.

##  INTRODUCTION

It is well-documented that retinopathy of prematurity (ROP) and its subsequent treatment significantly influence the development and biometrics of the eye ^[1]^. Studies have also confirmed that the incidence of myopia is notably higher in patients who have undergone ROP treatment.^[2]^ Biometric assessments indicate that previous ROP treatment leads to shallower anterior chambers and steeper corneas, thereby escalating the risk of both glaucoma and myopia. These structural modifications are hypothesized to stem from abnormal anterior segment development following laser treatment. Furthermore, research has shown that the degree of these structural changes is directly proportional to the size of the treatment area.^[3,4]^

Over the past decade, anti-vascular endothelial growth factor (anti-VEGF) agents have superseded laser treatment for ROP in many clinical scenarios. Given that the administration of anti-VEGFs in ROP has led to fewer ocular biometric alterations compared to earlier treatment methods (such as laser and cryotherapy), the ultimate refractive outcomes for these patients have also differed.^[2]^ Thus, eyes treated with anti-VEGF agents have exhibited a lower degree of myopia. Earlier studies comparing the long-term effects of laser and anti-VEGF agents have identified structural differences in both the anterior and posterior segments of the eye. It has been established that eyes treated with anti-VEGF agents possess thinner retinas, deeper anterior chambers, and thinner lenses compared to those treated with laser therapy.^[5]^ Different macular microstructures have been observed as a result of these two treatment methods. Numerous studies have compared the long-term effects of two ROP treatment methods, namely laser therapy and anti-VEGF injections, on the retinal structure, microvasculature, biometrics, and refractive error.^[1,6,7,8,9,10]^ Scheimpflug imaging systems have facilitated a comprehensive comparison of the long-term effects of these two ROP treatment methods on the development of the anterior segment and the structural and biometric characteristics of treated eyes. Consistently, previous findings have shown that eyes treated with anti-VEGF drugs undergo fewer changes in the anterior segment compared to those treated with laser.^[5,11]^ However, during our investigation, we noted a significant number of children with a history of ROP treatment who exhibited no substantial refractive errors, and their anterior segment measurements were comparable to those of non-ROP children. This observation led us to question whether all children with a history of ROP treatment should be viewed as a homogeneous group, or if they should be differentiated from those who exhibit ROP-associated myopia and biometric changes. As a result, this study was undertaken primarily to evaluate the hypothesis that the development of ROP-associated myopia and biometric changes in children with a history of ROP treatment are intrinsically linked. By investigating this relationship, we aimed to illuminate the subtle variations within this population and gain a deeper understanding of the interplay between ROP treatments, the development of myopia, and associated biometric modifications.

##  METHODS 

This comparative cross-sectional study was carried out at Rassoul Akram Hospital in Tehran, Iran, from February 2021 to April 2022, in strict adherence to the principles of the Declaration of Helsinki. The study re-evaluated children with a history of ROP who had been treated with either intravitreal bevacizumab (IVB) or laser therapy. Written informed consent was obtained from each participant's legal guardians. The study received approval from the Ethics Committee of the Iran University of Medical Sciences (code: IR.IUMS.REC.1399.246).

This study included children who were at least five years old and had a history of ROP treatment. All the enrolled children had been diagnosed with type 1 ROP in zone 2 and had undergone either laser or bevacizumab therapy. The severity of ROP cases was graded based on the highest level observed during the acute phase.^[12]^ Laser photocoagulations were performed under intravenous sedation. The avascular retina was treated using a laser with a wavelength of 810 nm. Typically, a 360º laser treatment was initiated with a low power setting of 300 milliwatts for 150 milliseconds in repeat mode, set at 250 milliseconds. The power settings were adjusted as needed to ensure the avascular retina was covered entirely with closely spaced laser spots. For intravitreal anti-VEGF treatment, the ophthalmologists used 0.625 mg bevacizumab (Avastin; Genentech, South San Francisco, California, USA), which was injected 1.5 millimeters posterior to the limbus using a 31G needle. After just a single therapy session, all individuals treated with either laser or anti-VEGF agent exhibited ROP regression.

Children who had a history of combined treatment (i.e., laser rescue treatment following anti-VEGF therapy), those who required any ocular surgery (e.g., glaucoma, cataract, or retinal surgery) after the initial ROP therapy, and those who progressed to ROP stage four or five were excluded from the study. Additionally, children who did not cooperate during ophthalmic imaging and participants with structural ocular abnormalities (e.g., retinal folds, macular dragging, or corneal scars) that interfered with appropriate fixation were also excluded. The control group consisted of full-term children of a similar age with myopic refraction (spherical equivalent) who had no history of ocular diseases or conditions.

### Ophthalmic Examination 

Both the case and control groups underwent a comprehensive ophthalmic evaluation, which included the measurement of the best-corrected visual acuity (BCVA) using a Snellen eye chart, slit-lamp biomicroscopy, and indirect ophthalmoscopy. Forty-five minutes after the administration of three tropicamide 1% eye drops (Sina Darou, Iran), spaced five minutes apart, cycloplegic refraction was performed using a kerato-refractometer (KR-800, Topcon, Tokyo, Japan). For statistical analysis, visual acuity was gauged using a standard E-chart, and the result was subsequently converted to logMAR. Myopia was defined as a spherical equivalent (SE) of ≤ –0.25 diopters (D), while high myopia was categorized as an SE of ≤ –5.00 D.^[13]^

### Ocular Biometric Evaluation

The optical biometric profiles of all children, including corneal curvature, anterior chamber depth (ACD), lens thickness (LT), and axial length (AL), were measured using IOL Master 700 (Carl Zeiss Meditec AG, Jena, Germany). Anterior segment imaging was conducted using the Pentacam device (Oculus Systems, Wetzlar, Germany). For each eye, parameters such as ACD, volume, angle, and corneal structure were meticulously recorded.

### Statistical Analysis

Variables were categorized into two types based on their association with the patient or the eye. Patient-associated variables such as age and gender were classified as patient-level variables, while those related to eye characteristics and biometric measurements were designated as eye-level variables.

To investigate the relationship between qualitative patient-level variables, we performed the chi-square test. For comparing the means of patient-level variables across groups, either an analysis of variance or an independent *t*-test was employed. Given that all eye-level variables were measured within paired eyes, it was necessary to consider the correlation between these paired eyes. To accommodate this correlation, the generalized estimating equation (GEE) method was applied. Statistical analyses were conducted using SPSS version 24.0 (IBM SPSS Statistics for Windows, Version 24.0. Armonk, NY: IBM Corp). *P* < 0.005 was considered statistically significant.

##  RESULTS

This study included a total of 31 patients: 14 in the IVB group (10 males and 4 females) and 17 in the laser-treated group (7 males and 10 females). Additionally, 13 individuals were recruited as a control group. All treated patients received identical bilateral treatment. The mean gestational age (GA) was 28.21 ± 2.32 weeks in the IVB group and 28.12 ± 1.9 weeks in the laser-treated group (*P *= 0.899). The mean birth weight (BW) was 1166.43 ± 395.17 grams in the IVB group and 1090.6 ± 253.7 grams in the laser-treated group (*P *= 0.520). At the time of examination, the mean age was 9.94 ± 2.60 years in the laser-treated group and 9.14 ± 1.65 years in the IVB group (*P *= 0.286). The two study groups had no significant differences regarding GA, BW, and age. The mean age of the participants in the control group was 9.08 ± 2.35 years, and no significant differences were observed in the subjects' ages across all three study groups [Table red3].

**Table 1:** Comparative analysis of refraction and ocular biometrics in eyes treated with laser and intravitreal bevacizumab (IVB)

	**IVB (** ***n*** ** = 28 eyes)**	**Laser (** ***n*** ** = 34 eyes) **	***P*** **-value**
Visual acuity (LogMAR)	0.12 ± 0.13	0.19 ± 0.15	0.114
Spherical equivalent (D)	–0.47 ± 2.48	–1.99 ± 4.8	0.224
Refraction cylinder (D)	–1.57 ± 1.02	–2.60 ± 2.60	0.061
Myopia	14/28 (50%)	18/34 (52.9%)	0.713
High myopia	0/28 (0.00%)	7/34 (20.6%)	< 0.001
Astigmatism	22/28 (78.6%)	27/34 (79.4%)	0.873
Corneal thickness (microns)	548.11 ± 31.90	547.59 ± 45.85	0.949 20% ${\;}^{*}$
	CI: 536.29 to 559.93	CI: 531.59 to 562.41	
Mean keratometric reading (D)	44.54 ± 2.05	46.22 ± 1.72 CI: 45.64 to 46.80	0.10 60% ${\;}^{*}$
	CI: 43.78 to 45.30	CI: 45.64 to 46.80	
Anterior chamber depth (mm)	3.02 ± 0.42	2.57 ± 0.37	0.001
	CI: 2.86 to 3.18	CI: 2.44 to 2.69	
Anterior chamber volume (µm^3^)	180.93 ± 29.99	141.00 ± 41.55	< 0.001
	CI: 170.85 to 191.01	CI: 127.03 to 154.97	
Lens thickness (mm)	3.48 ± 0.29	3.88 ± 0.32	< 0.001
	CI: 3.37 to 3.59	CI: 3.77 to 3.99	
Axial length (mm)	21.71 ± 1.24	21.94 ± 1.72	0.745 15% ${\;}^{*}$
	CI: 21.29 to 22.13	CI: 21.36 to 22.52	
D, diopters; IVB, intravitreal bevacizumab; LogMAR, logarithm of the minimum angle of resolution			
${\;}^{*}$ Power analysis of comparisons between groups			

**Table 2:** Association between refractive error and ocular biometric alterations in ROP subgroups

		**Total**	**IVB (** ***n*** ** = 28 eyes)**	**Laser (** ***n*** ** = 34 eyes)**	**Myopic (** ***n*** ** = 32 eyes)**	**Non-myopic (** ***n*** ** = 30 eyes)**
Mean keratometry reading	β	–0.19	0.39	–0.41	–0.40	0.13
	*P*-value	0.396	0.273	0.309	0.001	0.394
Axial length	β	–1.35	–0.87	–2.34	–0.57	–0.94
	*P*-value	0.001	0.174	< 0.001	0.048	0.003
Anterior chamber depth	β	3.18	–0.130	6.77	3.86	1.98
	p-value	0.024	0.559	< 0.001	< 0.001	0.334
Lens thickness	β	–3.10	–0.85	–3.93	–3.73	–2.21
	*P* -value	0.012	0.764	0.049	< 0.001	0.315
Corneal thickness	β	–0.01	–0.01	–0.02	–0.02	–0.01
	*P* -value	0.172	0.633	0.046	0.006	0.468
IVB, intravitreal bevacizumab						
β : regression coefficient from a multivariate regression analysis						

**Table 3:** Comparative analysis of ocular biometrics and refraction across study groups

	**Myopic ROP (** ***n*** ** = 32 eyes)**	**Control group (** ***n*** ** = 26 eyes)**	**Non-myopic ROP (** ***n*** ** = 30 eyes)**	***P*** **-value**	B
Age (yrs)	9.85 ± 2.08	9.08 ± 2.35	9.36 ± 2.21	0.212 ${\;}^{\#}$	–
Spherical equivalent (diopters)	–3.89 ± 3.27	–2.50 ± 160	1.65 ± 1.87	0.074 ${\;}^{*}$	–1.38
				< 0.001 ${\;}^{**}$	3.67
				< 0.001 ${\;}^{***}$	5.49
Mean keratometric reading (diopters)	46.31 ± 2.30	43.50 ± 1.71	45.73 ± 1.92	< 0.001 ${\;}^{*}$	2.89
				< 0.001 ${\;}^{**}$	2.48
				0.958 ${\;}^{***}$	0.03
Axial length (mm)	22.11 ± 1.64	23.49 ± 0.93	21.66 ± 1.16	< 0.001 ${\;}^{*}$	–1.50
				< 0.001 ${\;}^{**}$	–1.90
				0.048 ${\;}^{***}$	–0.86
Anterior chamber depth (mm)	2.68 ± 0.46	2.98 ± 0.44	2.92 ± 0.39	0.49 ${\;}^{*}$	–0.30
				0.425 ${\;}^{**}$	–0.11
				0.763 ${\;}^{***}$	–0.04
Lens thickness (mm)	3.78 ± 0.41	3.42 ± 0.15	3.58 ± 0.29	< 0.001 ${\;}^{*}$	0.39
				0.010 ${\;}^{**}$	0.20
				0.623 ${\;}^{***}$	0.05
Corneal thickness (microns)	552.53 ± 35.81	549.19 ± 25.37	563.73 ± 27.88	0.721 ${\;}^{*}$	3.56
				0.129 ${\;}^{**}$	13.27
				0.605 ${\;}^{***}$	2.28
${\;}^{\#}$ Comparison of study groups using ANOVA; ${\;}^{*}$ Myopic ROP vs control group using the GEE method; ${\;}^{**}$ Control group vs non-myopic ROP using the GEE method; ${\;}^{***}$ Myopic ROP vs non-myopic ROP using the GEE method					

### Refractive Outcome

The SE of the refractive error was –0.47 ± 2.48 in the IVB group and –1.99 ± 4.81 in the laser-treated group (*P *= 0.224) [Table 1]. In the IVB group, 50% (14/28) of the eyes were myopic, compared to 52.9% (18/34) in the laser-treated group. The prevalence of high myopia was significantly higher in the laser-treated group (0.00% vs 20.6%, *P* < 0.001) [Table 1].

The mean astigmatism was –2.60 ± 2.60 D in the laser-treated group and –1.57 ± 1.02 D in the IVB group (*P *= 0.061) [Table 1]. The prevalence of astigmatism (cylinder ≥ 1.00 D) was 79.4% (27/34) in the laser-treated group and 78.6% (22/28) in the IVB group. Among the patients receiving IVB, 42.85% (6/14) exhibited bilateral myopia, while only 14.28% (2/14) had unilateral myopia. In the laser-treated group, 47.06% (8/17) presented with bilateral myopia, and 11.76% (2/17) had unilateral myopic refraction. Additionally, the presence of asymmetric refractive error, defined by an SE difference of > 1.00 D, was observed in 21.43% (3/14) of patients in the IVB group and 70.59% (12/17) of patients in the laser-treated group.

### Comparison of Biometric Data Between IVB and Laser Groups

The mean AL of the eyes in the laser-treated group was 21.71 ± 1.24 mm, compared to 21.94 ± 1.72 mm in the IVB group (*P *= 0.745). The eyes treated with laser had a significantly shallower ACD and thicker lenses than those in the IVB group (*P *= 0.001) [Table 1]. They also had a significantly lower anterior chamber volume (141.00 ± 41.55 vs 180.93 ± 29.99, *P* < 0.001) [Table 1]. There were no significant differences in corneal thickness (CT) and mean keratometric readings (KR) between the two intervention groups (*P* = 0.949 and *P* = 0.10, respectively) [Table 1].

### Relationship Between Biometric Changes and Refractive Error

Table 2 shows that when all patients are considered together, changes in refractive error are not related to the mean K changes. It also indicates a positive relationship between SE and ACD, suggesting that shifts toward myopia are associated with a decrease in ACD. Additionally, there was a negative relationship between SE and both LT and AL, indicating that shifts toward myopia occur with an increase in lens thickness and axial length.

Interestingly, after the study groups were categorized based on their treatment modalities, the relationship between ocular biometrics and SE became more pronounced in the laser-treated group and less in the IVB group. We found a significant relationship between SE and AL, ACD, LT, and CT in the laser-treated group. Such a relationship was not observed in the IVB group. Furthermore, we divided the study population based on their refractive error state, regardless of their treatment modality. In the non-myopic group, only AL had a significant relationship with SE (*P *= 0.003) [Table 2], whereas all biometric parameters showed a significant relationship with SE in the myopic group [Table 2].

### Comparison Between Myopic ROP and Control Groups

Table 3 compares the biometric data between myopic children in the ROP and myopic control groups. The mean age and SE refraction did not significantly differ between the two groups. However, children in the ROP group had a significantly shorter AL (*P* < 0.001), shallower AC (*P* < 0.001), thicker lens (*P* < 0.001), and steeper cornea (*P* < 0.001). The difference in corneal thickness between the two groups was not statistically significant (*P *= 0.721). As demonstrated in Table 3, even though the myopic ROP patients had a significantly lower SE, a thicker lens, shallower AC, longer AL, and steeper corneas than non-myopic ROP patients, the only significant difference between their biometric data was in AL (*P *= 0.048).

In our comparative analysis, the results shed light on significant differences among the high myopic ROP group, controls (myopic non-ROP group), and myopic ROP group. While the AL measurements did not show statistically significant differences across the three groups (22.58 ± 1.46 for high myopic ROP patients), as reflected by *P*-values of 0.471 for the myopic ROP group and 0.281 for the control group, significant variations were observed in other ocular parameters. Specifically, the SE values of high myopic ROP patients (–9.32 ± 2.59) were notably lower compared to both the myopic ROP and control groups (both *P* < 0.001). Furthermore, a statistically significant difference was observed in ACD, with high myopic ROP patients exhibiting smaller values (2.17 ± 0.51) compared to the myopic ROP and control groups (both *P* < 0.001). Moreover, high myopic ROP patients displayed higher lens thickness values (4.19 ± 0.45 mm) compared to the other groups, with statistically significant differences observed (*P* < 0.001 for both myopic non-ROP and myopic ROP groups). Additionally, the corneal curvature values revealed steeper corneas in high myopic ROP patients (48.00 ± 1.19), with statistically significant differences noted when compared to both the myopic ROP and control groups (*P* < 0.05 for both comparisons).

##  DISCUSSION

Numerous studies have highlighted the increased prevalence of myopia among children who have undergone ROP treatment. The prevailing suggestion is that this myopic refractive error is primarily triggered by the biometric changes in the ocular anterior segment.^[3,14]^ Our study aligns with this notion, demonstrating that in children with a history of ROP treatment, changes in refraction are closely associated with alterations in AL, LT, and ACD. In other words, a higher degree of myopia in these children corresponds to thicker lenses, shallower anterior chambers, and longer axial lengths.

It is worth noting that our findings are inconclusive, as we have observed cases where children with a history of ROP treatment showed minimal changes in refraction and ocular biometry. In other words, these children exhibited refractive errors and ocular biometry similar to normal children's. As previously stated, in these children, refraction changes correlate with variations in AL, LT, and ACD. However, after dividing patients into myopic and non-myopic groups, we found that the relationships between refractive error and anterior segment biometric changes were only apparent in the myopic group. While refraction in non-myopic children was linked solely to changes in AL, myopic children showed changes in refractive error associated with alterations in AL, LT, ACD, and mean KR. This suggests that even with a history of treatment, a subset of ROP patients can achieve normal ocular development, as reported by Fieß et al.^[6]^ Unlike our study, their research included both treated and non-treated eyes.

To highlight the distinct biometric changes associated with myopia in children with a history of ROP, we included a myopic control group for comparative analysis. Our findings revealed significant differences between the myopic ROP group and the control group. Specifically, the myopic ROP group exhibited notably shorter AL, shallower AC, and thicker lenses. These unique findings suggest that the ocular biometry of myopic ROP children should be recognized as a distinct characteristic that differentiates them from non-myopic eyes with a history of ROP and myopic eyes without any history of ROP.

Our data further bolster previous research that underscores the differences between eyes treated with IVB and laser in refraction and biometric changes. While the prevalence of myopia was comparable in both groups, the presence of high myopia was exclusively observed in laser-treated eyes. This is most likely due to more significant developmental changes in the anterior segment that occur in laser-treated eyes during the first year of life.^[10]^ Additionally, we discovered that although the two groups had similar axial lengths, the anterior chamber was significantly shallower, and the lens was thicker in the laser-treated group. This finding aligns with previous reports comparing the long-term biometric effects of anti-VEGF and laser treatments.^[3,11]^ A significant difference between the two study groups was the correlation between changes in refractive error and biometric modifications. Contrary to the previous report by Yang et al,^[15]^ which attributed myopic refraction exclusively to changes in the anterior segment in children, our study suggests that axial length elongation also plays a significant role in the myopic shift (or reduced hyperopic refraction), particularly in the non-myopic group. This finding holds true even after adjusting for age. However, in contrast to the previous report by Gunay et al,^[16]^ we could not establish a significant correlation between refractive error and AL elongation in the IVB group, possibly due to the small sample size of our study. Nevertheless, our findings emphasize that changes in the anterior chamber remain the most influential factor in this context.

A significant limitation of this study was the relatively small sample size, particularly in the IVB-treated group. Consequently, we could not identify a significant correlation between refractive error and changes in ocular biometry within this group. Including a larger number of participants in future studies could provide more insightful observations on the evolution of ocular biometry and its relationship with refractive changes. Moreover, conducting longitudinal studies on each group would offer valuable data on the progression of ocular biometric changes and their correlation with refractive shifts. It is particularly interesting to determine the critical point at which these children start transitioning toward myopia. Additionally, it would be worthwhile to explore whether non-myopic children eventually shift toward myopia or maintain their non-myopic status. Further research is needed to address these points.

In summary, this study underscores the unique characteristics of myopic and non-myopic eyes that have undergone ROP treatment, and the results suggest that they should be considered distinct entities. While alterations in axial length primarily drove changes in refractive error in non-myopic ROP, the myopic ROP group demonstrated a stronger correlation with changes in the anterior segment. Furthermore, children with myopic ROP exhibited a variety of biometric changes compared to myopic children without a history of ROP treatment, further highlighting their unique nature. Therefore, we propose that children with ROP-related myopia should be acknowledged as a distinct subgroup within the larger category of children with myopic eyes.

##  Financial Support and Sponsorship

The Eye Research Center of the Iran University of Medical Sciences financially supported this study but was not involved in its design or execution.

##  Conflicts of Interest

None.
